# Can users trust AI doctors? Unpacking the dual credibility pathways linking AI chatbot use to AI health advice adherence intention

**DOI:** 10.3389/fpsyg.2026.1948888

**Published:** 2026-10-07

**Authors:** Zhanyou Wang, Jingru Wang, Dongmei Han, Liang Ma, Feifei Hao

**Affiliations:** 1 School of Business Administration, Shandong Management University, Jinan, China; 2 School of Labor Relations, Shandong Management University, Jinan, China; 3 School of Information Engineering, Shandong Management University, Jinan, China; 4 School of Management Science and Engineering, Shandong University of Finance and Economics, Jinan, China; 5 College of Traditional ChineseMedicine, Shandong University of Traditional Chinese Medicine, Jinan, China

**Keywords:** ability credibility, AI doctor utilization, AI health advice adherence intention, anxiety sensitivity, perceived honesty

## Abstract

With the rapid integration of artificial intelligence (AI) into healthcare, AI doctor chatbots have emerged as increasingly prevalent tools for health information acquisition and health management. However, existing research has paid insufficient attention to how AI doctor utilization is associated with AI healthcare users' credibility judgments and subsequent AI health advice adherence intention. To address this research gap, this study examines the relationship between AI doctor utilization and AI health advice adherence intention, with a particular focus on the mediating roles of perceived honesty and ability credibility, as well as the moderating role of anxiety sensitivity. A questionnaire survey was conducted among participants from various regions of China, and 224 valid responses were retained after data cleaning. SmartPLS 4.0 was used for data analysis. The results show that AI doctor utilization is significantly and positively associated with both perceived honesty and ability credibility, which, in turn, are positively associated with AI health advice adherence intention. Additionally, perceived honesty and ability credibility play mediating roles in the relationship between AI doctor utilization and AI health advice adherence intention. Regarding the moderating effect, anxiety sensitivity negatively moderates the relationship between perceived honesty and AI health advice adherence intention, while positively moderating the relationship between ability credibility and AI health advice adherence intention. These findings provide nuanced insights for designing anxiety-sensitive AI healthcare services by tailoring credibility cues to users' psychological characteristics and promoting appropriate reliance on AI-generated health advice.

## 1 Introduction

The integration of artificial intelligence (AI) into healthcare has revolutionized service delivery, with AI doctor chatbots emerging as pivotal tools for health information dissemination, preliminary diagnosis, and chronic disease management (Zhang et al., 2026a). In this study, AI doctor chatbots refer to AI-powered conversational systems that users interact with to obtain health information, discuss symptoms, receive preliminary health assessments, and obtain health-related recommendations. Rather than autonomous licensed physicians, these systems function as AI-based health-advice tools that support users' health-related decision making. The proliferation of AI medical tools introduces a new dynamic: while these technologies offer unprecedented accessibility and efficiency, their association with users' adherence intentions remains underexplored. Existing research has primarily focused on individual traits or organizational factors as drivers of AI health advice adherence intention (Li et al., 2026), overlooking how AI usage shapes cognitive perceptions and subsequent adherence intention (Dang and Liu, 2025). This gap highlights the need for empirical investigations into the mechanism linking AI doctor utilization to AI health advice adherence intention, with implications for understanding psychological processes underlying users' health-related decision-making in AI-enabled healthcare contexts.

First, existing research on AI health advice adherence intention has primarily focused on individual characteristics, organizational governance, and technical infrastructure, with relatively limited attention to the cognitive processes through which users evaluate AI-generated health advice (Ju et al., 2025; Liu et al., 2025). Although trust is widely recognized as an important antecedent of healthcare technology acceptance, it is often treated as a unidimensional construct. Such a treatment may obscure an important distinction in AI healthcare: users may evaluate not only whether an AI system is honest and acts without deception, but also whether it possesses sufficient technical competence to provide accurate and reliable health advice. These two credibility judgments may become particularly distinguishable in high-stakes healthcare contexts, where the consequences of technical errors, such as inaccurate assessments or recommendations, differ fundamentally from those associated with perceived insincerity. Information processing theory (IPT) provides a useful lens for understanding this distinction because it suggests that individuals interpret external information cues and form cognitive evaluations that guide subsequent behavioral responses (Kronlid et al., 2024). From this perspective, perceived honesty and ability credibility represent two distinct credibility judgments derived from users' evaluation of ethical and competence-related cues, respectively. However, little is known about whether these two judgments constitute distinct pathways through which AI doctor utilization is associated with users' adherence to AI-generated health advice. Addressing this question is also practically important because identifying how different credibility judgments relate to adherence can inform the design and effective application of AI healthcare tools (Chakraborty et al., 2023). Accordingly, our first research question asks: How do perceived honesty and ability credibility mediate the relationship between AI doctor utilization and adherence to AI-generated health advice?

Second, understanding these credibility pathways alone does not explain why the same credibility judgment may translate into different adherence decisions across users. Anxiety sensitivity, defined as an individual's tendency to interpret physical or health-related sensations as threatening, represents a particularly relevant boundary condition because it shapes how individuals respond to health-related information (Otto and Smits, 2018). Rather than treating anxiety sensitivity as a direct predictor of adherence, we conceptualize it as an emotional condition that alters how users translate different credibility judgments into behavioral decisions. IPT suggests that information processing is shaped not only by cognitive evaluations but also by individuals' emotional states, which may influence how different information cues are considered in decision making (Chen et al., 2023). In AI healthcare, this implies that anxiety-sensitive users may not simply become more or less trusting of AI overall; instead, anxiety sensitivity may condition the extent to which honesty-based and competence-based credibility judgments are associated with users' intentions to follow AI-generated health advice. Yet prior research has largely examined anxiety sensitivity as a direct predictor of health behavior or has overlooked its role in the transformation of credibility judgments into adherence (Otto and Smits, 2018). This leaves an important boundary condition unexplained: whether anxiety sensitivity changes the strength of the relationships between different credibility judgments and AI advice adherence intention. Accordingly, this study addresses the following research questions:

RQ1: How do perceived honesty and ability credibility mediate the relationship between AI doctor utilization and AI health advice adherence intention?
RQ2: How does anxiety sensitivity differentially shape the roles of honesty-based and competence-based credibility in users' adherence to AI-generated health advice?

To address these questions, this study draws on survey data from 224 AI healthcare users in China and employs SmartPLS 4.0 (SmartPLS GmbH, Germany) to test the proposed model. The findings reveal that AI doctor utilization is positively associated with AA through the mediating roles of both PH and AC. Additionally, AS negatively moderates the PH–AA relationship but positively moderates the AC–AA relationship, revealing an asymmetric pattern in how anxiety sensitivity conditions the two credibility pathways. These findings make two key theoretical contributions. First, this study advances this literature by distinguishing honesty-based and competence-based credibility judgments and revealing their distinct mediating roles in the relationship between AI doctor utilization and AI health advice adherence intention. This distinction provides a more fine-grained understanding of how users evaluate AI-generated health advice in high-stakes healthcare contexts. Second, this study identifies anxiety sensitivity as a theoretically meaningful boundary condition that differentially shapes the roles of PH and AC. This asymmetric moderation pattern is consistent with a differentiated credibility-processing perspective, suggesting that the relevance of honesty- and competence-based credibility judgments may vary across users with different levels of anxiety sensitivity. These findings extend the application of information processing theory by identifying anxiety sensitivity as a potential boundary condition that differentially shapes the relationships between distinct credibility judgments and adherence intention.

## 2 Theoretical background

### 2.1 Conceptual framework and key constructs

This study examines AI doctor utilization (AU) as the independent variable, AI health advice adherence intention (AA) as the dependent variable, perceived honesty (PH) and ability credibility (AC) as two mediating variables, and anxiety sensitivity (AS) as the moderating variable (Chen et al., 2021). AI doctor utilization refers to the extent to which individuals incorporate AI doctor chatbots into their health-related information seeking, consultation, decision support, and health-management activities. AA refers to an individual's self-reported willingness and intention to follow, implement, and continue acting upon health-related recommendations provided by AI doctors. It reflects an intended behavioral response to AI-generated health advice rather than objectively observed medical adherence behavior. Unlike traditional medical compliance, which typically concerns patients' adherence to physician-prescribed treatment regimens, AA captures adherence to health recommendations generated by AI systems without direct physician involvement. Accordingly, it represents a distinct form of adherence in AI-enabled healthcare rather than conventional clinical compliance. PH and AC represent two distinct credibility judgments regarding AI doctor chatbots (Jing, 2025). PH reflects users' perceptions that AI-generated health information is honest, transparent, and not intentionally misleading, whereas AC reflects their evaluations of the AI system's professional competence, accuracy, and reliability of medical knowledge. Although related, these two judgments capture different dimensions of credibility: PH concerns the ethical and intentional basis of AI-generated information, whereas AC concerns its technical and competence-based basis. This distinction is particularly relevant in high-stakes healthcare contexts because users may perceive an AI system as honest while remaining uncertain about its technical competence, or consider it technically capable while questioning the transparency or integrity of its recommendations. AS refers to an individual emotional trait characterized by the tendency to interpret physical discomfort and health-related sensations as threatening and to respond with heightened anxiety toward potential health risks (Khoury et al., 2021). Prior research suggests that interactions with technology are associated with users' cognitive evaluations of technological attributes (Liu Y. X. et al., 2026), which may subsequently relate to their behavioral responses in healthcare contexts. Drawing on information processing theory, this study proposes that AI doctor utilization is associated with AA through two credibility pathways—PH and AC. Importantly, AS is conceptualized not as a direct determinant of AA, but as a boundary condition that differentially shapes how these two credibility judgments are translated into adherence decisions (Lebowitz et al., 2015). Thus, the proposed framework integrates cognitive credibility evaluation with individual emotional characteristics to explain both how and under what conditions AI doctor utilization is associated with AI health advice adherence intention.

### 2.2 Information processing theory

Information Processing Theory (IPT) provides the theoretical foundation for this study. IPT suggests that individuals' behavioral judgments and decisions are shaped by how they attend to, interpret, and evaluate information from their external environment (Kelemen et al., 2026). The theory generally conceptualizes information processing as involving several stages, including attention, encoding and comprehension, inference, and response, through which external information is transformed into cognitive judgments and subsequent behavioral responses. Accordingly, IPT offers a useful framework for explaining how users' interactions with AI healthcare systems are associated with the formation of credibility judgments and, ultimately, their responses to AI-generated health advice. IPT is particularly relevant to the present study for two reasons.

First, AI doctor utilization exposes users to health-related information that requires cognitive evaluation before adherence decisions are made (Qin et al., 2022). Rather than examining the entire information-processing sequence, this study focuses on the credibility inference stage of IPT, distinguishing two credibility judgments: perceived honesty (PH), reflecting the honesty and transparency of AI-generated information, and ability credibility (AC), reflecting its competence, accuracy, and reliability. This distinction provides a more fine-grained explanation of how users evaluate AI-generated health information. Second, IPT suggests that emotional characteristics can shape how cognitive evaluations are translated into behavioral responses (Choi et al., 2026). Accordingly, anxiety sensitivity is conceptualized as a boundary condition that may differentially shape the relationships between honesty- and competence-based credibility judgments and adherence intention. Together, these arguments provide the theoretical basis for the study's dual credibility pathways and the differential moderating role of anxiety sensitivity.

Accordingly, the application of IPT in this study can be summarized as AI doctor utilization → credibility evaluation → AI health advice adherence intention, with anxiety sensitivity conditioning the relationship between credibility evaluation and adherence (Al-Qudimat et al., 2025). More specifically, AI doctor utilization provides the informational context in which users form two distinct credibility judgments—PH and AC—which are subsequently associated with their willingness to follow AI-generated health advice. The theoretical contribution lies in differentiating honesty- and competence-based credibility judgments and identifying anxiety sensitivity as a boundary condition that may differentially shape their relationships with adherence intention. Accordingly, IPT is used as a theoretical lens for interpreting the observed relationships rather than as a directly tested model of attention allocation, cue salience, or information weighting. This framework therefore explains how AI doctor utilization is associated with AI health advice adherence intention through distinct credibility pathways and why the strength of these relationships may vary across users.

### 2.3 AI healthcare adoption and use

Existing research on AI healthcare adoption and use has primarily examined the factors that shape users' acceptance, continued use, and behavioral responses to AI-enabled healthcare services (Bansal et al., 2026; Maheux et al., 2026) (Table 1). Studies have shown that users' responses to AI healthcare technologies are influenced not only by the functional characteristics of AI systems but also by psychological, social, and institutional factors. For example, perceived intrusiveness and individuation can shape patient compliance with AI vs. human doctors (Chen et al., 2021), while trust, physician endorsement, explainability, and ethical considerations are important for the acceptance of AI healthcare agents (Jing, 2025). Other studies have highlighted the roles of utilitarian and hedonic values, satisfaction, IT identity, and social norms in shaping continued chatbot use (Esmaeilzadeh et al., 2025), as well as the importance of legal–ethical safeguards, bias control, and human oversight in public healthcare applications (Khalique et al., 2026). More recent research further suggests that perceived intelligence and anthropomorphism can facilitate healthcare chatbot adoption through usefulness and trust-related perceptions (Li et al., 2026), while users' risk appraisal and the informational and emotional support provided by AI agents also shape their evaluations and behavioral responses (Yang et al., 2026; Zhang et al., 2026a). Collectively, this literature provides substantial insight into why users adopt, accept, and continue to use AI healthcare technologies. However, it remains less clear how users' actual utilization of AI doctors is translated into their willingness to follow AI-generated health advice. In particular, existing adoption-oriented research provides limited explanation of the credibility inference process after AI use (Springer et al., 2023), including whether users distinguish between honesty-based ethical credibility and competence-based technical credibility when deciding whether to rely on AI-generated health recommendations. Addressing this unresolved issue requires moving beyond the question of whether users adopt AI healthcare technologies toward understanding how AI doctor utilization is associated with subsequent adherence through distinct credibility judgments.

**Table 1:** AI use and user behavior in healthcare literature.

References	Use dimension	Mediator variable	Moderator variable	Dependent variable	Conclusion
Chen et al. (2021)	Healthcare Chatbot (AI/human doctor)	Perceived intrusiveness	Individuation (present/absent)	Patient compliance	AI doctor individuation reduces compliance; human doctor individuation improves compliance.
Jing (2025)	AI healthcare agents	Patient trust	Physician endorsement	Acceptance and adoption of AI healthcare agents	Explainability, ethics, and physician endorsement boost trust and adoption.
Esmaeilzadeh et al. (2025)	Hedonic values Utilitarian values of AI-based chatbots	Satisfaction, IT identity	Social norms associated with AI	Intention to explore new features, WOM Continued intention to use	Utilitarian and hedonic values positively influence user satisfaction with wellbeing outcomes. Satisfaction, in turn, shapes IT identity, IT identity positively influences behavioral intentions, including exploring new features, word of mouth, and continued use of AI chatbots.
Khalique et al. (2026)	AI in Indian public healthcare delivery	Legal-ethical compliance & audit	Data bias, policy, human oversight	Quality, equity, accessibility of care	AI improves Indian healthcare but needs strict legal-ethical rules, bias control, and human supervision.
Li et al. (2026)	Perceived intelligence and perceived anthropomorphism	Perceived usefulness, competence trust, and benevolence trust.	N/A	users' intention to adopt healthcare chatbots	Perceived intelligence and perceived anthropomorphism enhance users' intention to adopt healthcare chatbots through perceived usefulness, competence trust, and benevolence trust.
Yang et al. (2026)	AI Risk Appraisal	Problem-focused adaptation, Emotion-focused adaptation	N/A	Moral Risks Perception, Usage Intention	While LLMs achieve qualitative performance comparable to humans, they underperform in quantitative contexts due to repetitive responses, incomplete responses, confusing responses, and short-lived prompts
Zhang et al. (2026a)	Service agent (AI vs. Human)	Information support, Emotional support	Response length	Patient trust	Compared to AI doctors, patients exhibit higher levels of trust in human doctors across all three dimensions. Furthermore, informational support and emotional support mediate the relationship between service agent types (AI vs. human) and patient trust

## 3 Hypothesis development

Drawing on Information Processing Theory and prior research on AI-enabled healthcare (Esmaeilzadeh et al., 2025), this study examines the relationships among AI doctor utilization, perceived honesty, ability credibility, anxiety sensitivity, and AI health advice adherence intention. Specifically, it distinguishes honesty-based and competence-based credibility pathways and introduces anxiety sensitivity as a boundary condition that may differentially shape the translation of these credibility judgments into adherence. The following sections develop the hypotheses underlying these dual credibility pathways and the differential moderating role of anxiety sensitivity.

### 3.1 AI doctor utilization, perceived honesty and ability credibility

Information Processing Theory suggests that individuals form cognitive evaluations by processing information cues encountered through interactions with external objects and systems (Choi et al., 2026). In AI-enabled healthcare, interactions with AI doctor chatbots expose users to health information, recommendations, and system responses that provide cues for evaluating the credibility of the technology. As users engage more extensively with AI doctors, they have greater opportunities to evaluate both the ethical and technical attributes of AI-generated health information, thereby forming differentiated credibility judgments. Perceived honesty (PH) reflects users' judgment that AI doctor chatbots provide truthful, transparent, and non-deceptive health information while taking users' interests into account (Basharat and Shahid, 2026). From an information-processing perspective, repeated interactions provide users with opportunities to assess the consistency, transparency, and integrity of AI-generated information. When the information provided across interactions is perceived as consistent and transparent and does not appear intentionally misleading, users are more likely to develop favorable honesty-based credibility judgments. Thus, greater AI doctor utilization is expected to be positively associated with perceived honesty. Accordingly, we propose:

**H1:** AI doctor utilization is positively associated with perceived honesty.

Ability credibility (AC), in contrast, reflects users' evaluations of the professional competence, accuracy, and reliability of AI doctor chatbots. Through continued interactions, users are exposed to cues regarding the system's ability to respond to health-related questions, provide relevant recommendations, and generate medically useful information. Such experiences provide a basis for evaluating whether the AI system demonstrates sufficient technical competence and informational reliability (Hao and Li, 2025). As users engage more extensively with AI doctors, they have more opportunities to evaluate these competence-related cues and develop corresponding judgments regarding the system's ability. Therefore, greater AI doctor utilization is expected to be positively associated with ability credibility. Accordingly, we propose:

**H2:** AI doctor utilization is positively associated with ability credibility.

### 3.2 Perceived honesty, ability credibility and AI health advice adherence intention

From an information-processing perspective, users' decisions to follow AI-generated health advice depend on how they cognitively evaluate the credibility of the information source (Koyra et al., 2026). In AI-enabled healthcare, perceived honesty (PH) and ability credibility (AC) represent two distinct credibility judgments—honesty-based and competence-based credibility—that may shape users' willingness to follow AI-generated health advice (Özel, 2025). Because these judgments reflect different evaluations of AI healthcare systems, they may provide cognitive bases for AI health advice adherence intention. PH reflects users' perceptions that AI doctor chatbots provide truthful, transparent, and non-deceptive information while taking users' interests into account. When users perceive AI-generated health information as honest and transparent, uncertainty about the intentions and integrity of the system is reduced, making them more willing to accept and follow its recommendations. Conversely, doubts about the honesty or transparency of AI-generated information may increase skepticism and reduce willingness to rely on such advice (Dahka and Koivumäki, 2026). Thus, honesty-based credibility provides an important cognitive basis for users' adherence decisions. Accordingly, we propose:

**H3:** Perceived honesty is positively associated with AI health advice adherence intention.

AC reflects users' evaluations of the professional competence, accuracy, and reliability of AI doctor chatbots. In healthcare contexts, following AI-generated advice requires users to judge whether the system is sufficiently capable of providing reliable and useful health information. When users perceive an AI doctor as competent and its recommendations as accurate and reliable, they are more likely to regard its advice as credible and useful and, consequently, to follow that advice (Liu Z. K. et al., 2026). Thus, competence-based credibility provides a distinct cognitive basis for AI health advice adherence intention. Accordingly, we propose:

**H4:** Ability credibility is positively associated with AI health advice adherence intention.

### 3.3 Mediating roles of perceived honesty and ability credibility

Building on the preceding hypotheses, Information Processing Theory suggests that external information stimuli are associated with behavioral responses through individuals' cognitive evaluations. In AI-enabled healthcare, AI doctor utilization provides users with information and interaction experiences from which they form credibility judgments, while these judgments provide a cognitive basis for deciding whether to follow AI-generated health advice. Accordingly, perceived honesty (PH) and ability credibility (AC) may serve as two cognitive pathways linking AI doctor utilization to AI health advice adherence intention. Specifically, greater AI doctor utilization provides users with more opportunities to evaluate the honesty, transparency, competence, and reliability of AI-generated health information. These evaluations may subsequently shape users' willingness to follow AI-generated recommendations (Lei et al., 2026). PH represents an honesty-based pathway, through which perceptions of truthful and transparent information reduce skepticism toward AI-generated advice and facilitate its acceptance. In contrast, AC represents a competence-based pathway, through which perceptions of professional competence, accuracy, and reliability strengthen users' confidence in the usefulness of AI-generated advice. Thus, rather than relying on a single generalized trust mechanism, AI doctor utilization may be associated with AI health advice adherence intention through two distinct credibility pathways. Accordingly, we propose:

**H5:** Perceived honesty mediates the relationship between AI doctor utilization and AI health advice adherence intention.

**H6:** Ability credibility mediates the relationship between AI doctor utilization and AI health advice adherence intention.

### 3.4 Moderating role of anxiety sensitivity

Information Processing Theory suggests that emotional characteristics can shape how individuals translate cognitive evaluations into behavioral responses. Anxiety sensitivity, which reflects the tendency to interpret physical or health-related sensations as threatening, is particularly relevant in health-related decision-making. Rather than directly determining AI health advice adherence intention, AS may function as a boundary condition that alters the relative importance of different credibility judgments when users decide whether to follow AI-generated health advice (Cheney et al., 2025). Accordingly, we propose that AS differentially moderates the relationships of perceived honesty and ability credibility with AI health advice adherence intention, producing a pattern consistent with differential credibility weighting.

For PH, we propose a negative moderating effect of AS between perceived honesty and AI health advice adherence intention. PH provides users with reassurance that AI-generated information is honest, transparent, and non-deceptive. However, individuals with higher AS are more sensitive to potential health threats and uncertain outcomes (Wong et al., 2014). Under heightened health-related concern, honesty alone may become less sufficient as a basis for adherence because truthful intentions do not necessarily guarantee the accuracy or effectiveness of health recommendations. Consequently, among high-AS users, the positive relationship between perceived honesty and AI health advice adherence intention may become weaker. In contrast, among individuals with lower AS, health-related concerns may exert less influence on their responses to AI-generated advice, allowing the positive relationship between PH and adherence intention to remain relatively stronger. Therefore, we propose:

**H7:** Anxiety sensitivity negatively moderates the relationship between perceived honesty and AI health advice adherence intention.

For AC, we propose the opposite pattern. Individuals with higher AS have heightened concerns about health risks and therefore a stronger need for reliable and professionally competent health information. Under such conditions, competence-related evaluations—including perceived accuracy, expertise, and reliability—may become more relevant to adherence decisions under conditions of heightened health-related uncertainty. Thus, when AC is high, anxiety-sensitive users may be particularly likely to regard AI-generated advice as sufficiently reliable to follow. By contrast, among users with lower AS, competence-related credibility may have a comparatively weaker association with adherence intention because health-related uncertainty may exert less influence on their responses to AI-generated advice (Knapp et al., 2020). Accordingly, AS is expected to strengthen the positive relationship between AC and adherence. Therefore:

**H8:** Anxiety sensitivity positively moderates the relationship between ability credibility and AI health advice adherence intention.

Based on Information Processing Theory and the preceding hypotheses, this study develops an integrated research model linking AI doctor utilization, credibility judgments, anxiety sensitivity, and AI health advice adherence intention. The model proposes two credibility pathways, in which perceived honesty and ability credibility mediate the relationship between AI doctor utilization and adherence. It further incorporates anxiety sensitivity as a boundary condition that differentially shapes these two pathways, reflecting a pattern consistent with differential credibility weighting. The proposed research model is presented in Figure 1.

**Figure 1:**
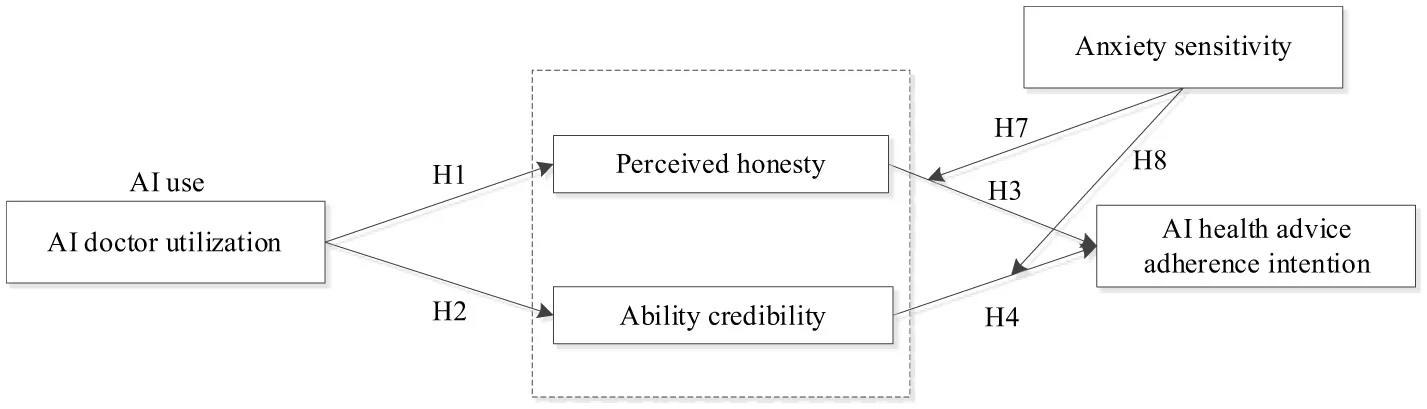
Research model.

## 4 Research methods

### 4.1 Measures

In this study, a questionnaire was employed to gather data. Each measurement item was sourced from prior literature (see Table 2). AI doctor utilization (AU) included four items adapted from Ma et al. (2024) and Ayo-Ajibola et al. (2024). Anxiety sensitivity (AS) was measured using four items adapted from the physical-sensation-related dimension of the Anxiety Sensitivity Index-3 (ASI-3) developed by Taylor et al. (2007). The selection of these items was guided by the conceptual relevance of anxiety sensitivity to health-related decision-making. Specifically, we focused on items capturing individuals' heightened sensitivity to physical sensations and concerns about the potential negative consequences of such sensations, which are particularly relevant when evaluating health-related information and advice. The items were contextually adapted to the AI healthcare setting while retaining the underlying meaning of anxiety sensitivity. Importantly, the items focus on individuals' reactions to and concerns about physical sensations rather than worry about a specific disease, persistent preoccupation with having an illness, or generalized health threats. Therefore, the measure is conceptualized as capturing the physical-sensation-related aspect of anxiety sensitivity in a health-related context rather than health anxiety *per se*. Given this context-specific adaptation, the four-item measure should not be interpreted as representing the full multidimensional ASI-3. Ability credibility (AC) consisted of four items taken from Mcknight et al. (2002), while perceived honesty (PH) included four items sourced from Gefen et al. (2003). AI health advice adherence intention (AA) was assessed with four items drawn from Bhattacherjee (2001). In particular, the original adherence-related items were adapted to reflect users' self-reported willingness and tendency to follow AI-generated health advice rather than conventional medication or physician-prescribed treatment adherence. Importantly, AI doctor utilization is conceptually distinct from AI health advice adherence intention. AI doctor utilization captures the extent to which individuals incorporate AI doctors into health-related activities, including obtaining health information, seeking consultation, considering AI-generated suggestions as informational inputs, and using AI doctors as reference sources for health management. In contrast, AI health advice adherence intention captures users' willingness to follow or implement the recommendations generated by AI doctors. Thus, considering AI-generated advice during decision-making or using it as a reference source does not necessarily imply accepting or acting upon that advice. A user may frequently consult an AI doctor and consider its suggestions while ultimately deciding not to follow them. Accordingly, the two constructs represent different stages of AI-enabled health decision-making: utilization reflects engagement with AI-generated information, whereas adherence reflects the subsequent acceptance and implementation of that information.

**Table 2:** Measurement items.

Constructs	Items measured
AI doctor utilization (AU)	AU1. I frequently use AI doctor chatbots to obtain health information
	AU2. I consult AI doctor tools when encountering health issues
	AU3. I use information provided by AI doctor chatbots as a reference when making health-related decisions.
	AU4. I use AI doctor chatbots as an important source of reference information for health management.
Anxiety sensitivity (AS)	AS1. I tend to become anxious when I experience physical discomfort.
	AS2. I tend to react more strongly than others to anxiety-related physical sensations.
	AS3. I tend to become concerned about even minor physical sensations.
	AS4. I tend to worry that unusual physical sensations may indicate something is wrong with my health
Ability credibility (AC)	AC1. I believe AI doctors possess sufficient professional competence
	AC2. I trust that AI doctors can provide accurate health advice
	AC3. I think AI doctors' diagnostic capabilities are trustworthy
	AC4. I consider AI doctors reliable in terms of medical knowledge
Perceived honesty (PH)	PH1. I believe AI doctors are honest when providing information
	PH2. I think AI doctors will not intentionally mislead users
	PH3. I feel the information provided by AI doctors is transparent PH4. I trust that AI doctors prioritize users' interests
AI health advice adherence intention (AA)	AA1. I am willing to follow health advice provided by AI doctors
	AA2. I intend to take actions in accordance with health advice provided by AI doctors.
	AA3. I intend to implement health recommendations provided by AI doctors.
	AA4. I am willing to continue following health guidance provided by AI doctors in the future.

All the aforementioned items were rated on a seven-point Likert scale, ranging from 1 (strongly disagree) to 7 (strongly agree). All measurement items were adapted from established scales and contextualized to the AI healthcare setting. Because the survey was administered in Chinese, the original English items were translated into Chinese and then back-translated into English to ensure semantic equivalence. The adapted questionnaire was subsequently reviewed by experts familiar with AI healthcare and information systems research to assess content validity and contextual appropriateness. A pretest was then conducted to evaluate the clarity, comprehensibility, and wording of the items. Based on the feedback, minor wording adjustments were made before the formal survey.

### 4.2 Data collection

In this study, participants from various regions of China (primarily Shandong Province, with additional samples from Beijing, Chongqing, Guangdong, and other regions) were recruited as respondents, and data were collected via WeChat, a widely used social media and online questionnaire distribution platform in China—this platform is frequently adopted in domestic social science research for its broad user reach and reliable data transmission. All participants provided informed consent prior to participation. For participants under the age of 18, consent was obtained from a parent or legal guardian, together with assent from the minor participant, in accordance with the approved ethical protocol. Specifically, the questionnaire links were shared through WeChat, and respondents submitted their answers voluntarily.

Data were collected between January 1 and January 30, 2026. A total of 292 questionnaires were initially received. Following the data-cleaning procedure, 68 responses were excluded: 25 due to response times below 90 s, 18 that failed the attention-check items, 15 showing straight-line/patterned response patterns, and 10 with excessive missing data. The final analytical sample consisted of 224 respondents. The final sample therefore represents general public users with prior experience using AI-based health-advice chatbots rather than a clinical patient population. Although participants were recruited from several regions of China, the sample was geographically concentrated in Shandong Province. Moreover, only five participants were under 18 years of age. Accordingly, the findings should be interpreted as reflecting AI health advice adherence intentions among general public users of AI-based health-advice chatbots, rather than actual adherence behavior among clinical patients.

To ensure that respondents had sufficient experiential basis to evaluate AI-based health-advice systems, participants were first screened for prior use of AI chatbots for health-related purposes. Only respondents who reported having used such systems to obtain health information, consult health-related problems, or seek health recommendations were eligible to complete the questionnaire. Participants were instructed to answer all subsequent questions based on their actual prior experiences with AI-based health-advice chatbots. To assess whether the sample size was adequate for testing the proposed model, we conducted a statistical power analysis using G^*^Power. The analysis was based on the endogenous construct with the largest number of predictors in the structural model. Assuming a medium effect size (*f*^2^ = 0.15), a significance level of α = 0.05, and statistical power of 0.80, the minimum required sample size was 98. The final sample of 224 respondents exceeded this requirement, suggesting that the sample size was adequate for detecting medium-sized structural relationships in the proposed model. Nevertheless, we acknowledge that a larger sample would provide greater precision, particularly for detecting relatively small interaction effects and assessing the replicability of the findings.

Table 3 presents the descriptive statistics of the participants' demographic characteristics (such as age, gender, education level, occupation, and income). We acknowledge that the sample consists primarily of general public users rather than clinically diagnosed users with chronic conditions, which limits the direct generalizability of findings to real-world treatment adherence scenarios; however, this sample is appropriate for an initial examination of users' credibility evaluations associated with AI advice acceptance, and future research should replicate findings in patient populations. The sample includes a relatively high proportion (33.93%) of participants with senior middle school education or below, which may affect generalizability to highly educated populations; however, this composition also provides insights into AI medical tool acceptance among less-educated users, a previously understudied group.

**Table 3:** Descriptive statistics of respondent characteristics.

Demographic variable		Size	%
Gender	Male	97	43.30
	Female	127	56.70
Age	< 18 years old	5	2.23
	18–30 years old	41	18.30
	31–40 years old	39	17.41
	41–50 years old	103	45.98
	>50 years old	36	16.07
Education	Senior middle school or below	76	33.93
	Junior college	62	27.68
	Bachelor's degree	62	27.68
	Master's degree or above	24	10.71
Income	Student, no income	23	10.27
	< = 5,000	85	37.95
	5,001–8,000	46	20.54
	8,001–10,000	22	9.82
	10,001–15,000	14	6.25
	15,001–20,000	8	3.57
	>20,000	26	11.61
Professional status	Employees of the enterprise	33	14.73
	Staff of public institutions	63	28.13
	Freelancer	40	17.86
	Entrepreneur	13	5.80
	Waiting for employment or unemployment	28	12.50
	Other	47	20.98

## 5 Data analysis and results

### 5.1 Measurement model

This study used SmartPLS 4.0 to evaluate the measurement model, encompassing reliability, convergent validity, and discriminant validity. For reliability assessment, Cronbach's alpha, composite reliability (CR), and average variance extracted (AVE) were adopted. As displayed in Table 4, Cronbach's alpha values range from 0.813 to 0.934, all exceeding the 0.7 threshold; composite reliability (CR) ranges from 0.877 to 0.953, which is above the 0.7 cutoff; and the average variance extracted (AVE) ranges from 0.641 to 0.836, surpassing the 0.5 threshold. These findings collectively indicate that all constructs demonstrate satisfactory reliability.

**Table 4:** Descriptive statistics and inter-construct correlations.

Items	Alpha	rho_A	CR	AVE	AU	AA	PH	AS	AC
AU	0.895	0.895	0.927	0.760	0.872				
AA	0.931	0.931	0.951	0.829	0.675	0.910			
PH	0.888	0.890	0.922	0.748	0.627	0.779	0.865		
AS	0.813	0.814	0.877	0.641	0.518	0.555	0.531	0.801	
AC	0.934	0.936	0.953	0.836	0.731	0.804	0.731	0.555	0.914

AI doctor utilization (AU); AI health advice adherence intention (AA); perceived honesty (PH); anxiety sensitivity (AS); ability credibility (AC).

Convergent validity and discriminant validity were confirmed through factor loadings and the square root of AVE, respectively. As presented in Table 5, all item factor loadings are above 0.5 (most exceeding 0.7) at the significance level of *p* < 0.01, verifying good convergent validity. To further confirm discriminant validity following contemporary guidelines, the square root of AVE for each construct (diagonal values in Table 4) is greater than its inter-construct correlation coefficients (off-diagonal values), which establishes adequate discriminant validity. All VIF values were less than 3, indicating no multicollinearity. The HTMT criterion provides a more robust test of discriminant validity, and all HTMT values are below 0.9 (Table 6), confirming that discriminant validity is acceptable (Zhang et al., 2026b). Furthermore, bootstrapped HTMT inference showed that the 95% confidence intervals for the AC–AA and PH–AA relationships did not include 1.00, providing additional evidence that the constructs are empirically distinguishable. Conceptually, PH, AC, and AA represent distinct psychological constructs despite their relatively strong associations. PH and AC capture users' evaluations of the AI doctor as an information source: PH reflects perceived ethical integrity, honesty, and transparency, whereas AC reflects perceived competence, accuracy, and technical reliability. In contrast, AA captures users' intended behavioral response to AI-generated health advice, namely their willingness and intention to follow and implement such recommendations. Thus, credibility judgments concern evaluations of the advice source, whereas adherence intention concerns users' prospective behavioral responses to the advice. Strong associations between these constructs are theoretically expected because favorable credibility judgments may facilitate adherence intention, but such associations do not imply conceptual equivalence.

**Table 5:** Cross loadings.

Items	AU	AA	PH	AS	AC
AC1	0.636	0.703	0.618	0.552	0.878
AC2	0.712	0.734	0.668	0.508	0.945
AC3	0.666	0.732	0.654	0.477	0.928
AC4	0.657	0.771	0.732	0.495	0.906
AS1	0.451	0.459	0.442	0.814	0.428
AS2	0.382	0.461	0.474	0.782	0.406
AS3	0.395	0.415	0.362	0.757	0.420
AS4	0.427	0.439	0.417	0.848	0.525
AU1	0.868	0.552	0.518	0.449	0.618
AU2	0.881	0.552	0.534	0.384	0.622
AU3	0.880	0.620	0.542	0.436	0.649
AU4	0.858	0.625	0.589	0.529	0.656
AA1	0.603	0.876	0.712	0.471	0.751
AA2	0.613	0.937	0.696	0.519	0.751
AA3	0.604	0.928	0.696	0.541	0.743
AA4	0.639	0.900	0.735	0.488	0.680
PH1	0.587	0.695	0.877	0.434	0.713
PH2	0.536	0.606	0.870	0.459	0.588
PH3	0.487	0.658	0.871	0.428	0.548
PH4	0.554	0.727	0.842	0.513	0.667

**Table 6:** HTMT results.

Items	AA	AC	AS	AU	PH
AA					
AC	0.861				
AS	0.637	0.638			
AU	0.739	0.798	0.605		
PH	0.854	0.798	0.622	0.7	

Because all focal variables were collected from the same respondents using self-reported measures, we took both procedural and statistical steps to assess the potential influence of common method bias (CMB). Procedurally, respondents were assured of anonymity and confidentiality, and the questionnaire emphasized that there were no right or wrong answers to reduce evaluation apprehension. Statistically, Harman's single-factor test showed that the first unrotated factor accounted for less than 50% of the total variance. Given the limitations of Harman's test, we further assessed common method variance using the full collinearity approach (Ma and Zhang, 2025). The resulting VIF values ranged from 1.987 to 2.802, all below the recommended threshold of 3.3. Taken together, these diagnostics suggest that severe common method variance is unlikely to dominate the observed relationships. Nevertheless, because the data were collected from the same respondents at a single point in time, common method variance cannot be completely ruled out.

### 5.2 Structural model

The structural model was assessed using inner VIF values, R^2^ and adjusted R^2^, effect sizes (f^2^), and predictive relevance measures. Bootstrapping was used to assess the significance and confidence intervals of the structural relationships. For the interaction effects, effect sizes and conditional effects at different levels of anxiety sensitivity were additionally examined. The model achieved a good fit with SRMR = 0.067 (< 0.08). The R^2^ of AI health advice adherence intention was 0.725, suggesting acceptable explanatory power.

Predictive relevance was further assessed using PLSpredict. At the latent-variable level, the Q^2^_predict values were 0.480 for AA, 0.530 for AC, and 0.386 for PH, with corresponding RMSE values of 0.728, 0.691, and 0.792 and MAE values of 0.577, 0.523, and 0.600, respectively. At the indicator level, all Q^2^_predict values were positive, ranging from 0.224 to 0.502, indicating predictive relevance across all endogenous indicators. To provide a more stringent assessment of out-of-sample predictive performance, we further compared the indicator-level RMSE values of the PLS-SEM model with those of the linear model (LM) benchmark. As shown in Table 7, PLS-SEM produced lower RMSE values than LM for 4 of the 12 indicators (AA1, AA2, AC3, and PH1), whereas LM produced lower RMSE values for the remaining eight indicators. Thus, although the positive Q^2^_predict values indicate predictive relevance, the comparison with the LM benchmark reveals mixed and relatively limited out-of-sample predictive performance. Accordingly, the model should not be interpreted as demonstrating uniformly superior predictive accuracy.

**Table 7:** PLSpredict assessment.

Indicator	Q^2^_predict	PLS-SEM RMSE	LM RMSE
AA1	0.376	1.200	1.217
AA2	0.398	1.118	1.126
AA3	0.394	1.106	1.100
AA4	0.416	1.118	1.116
AC1	0.399	1.248	1.148
AC2	0.502	1.196	1.165
AC3	0.438	1.184	1.203
AC4	0.425	1.181	1.180
PH1	0.335	1.246	1.249
PH2	0.279	1.276	1.272
PH3	0.224	1.264	1.257
PH4	0.300	1.291	1.258

The results of the structural model analysis are presented in Figure 2. First, AI doctor utilization is significantly and positively associated with PH (β = 0.627, *t* = 13.430, *p* < 0.001), thereby supporting Hypothesis 1. Second, AI doctor utilization is significantly and positively associated with AC (β = 0.731, *t* = 20.211, *p* < 0.001), which supports Hypothesis 2. Third, PH is significantly and positively associated with AA (β = 0.412, *t* = 6.801, *p* < 0.001), thus Hypothesis 3 is supported. Fourth, AC is significantly and positively associated with AA (β = 0.503, *t* = 7.656, *p* < 0.001), supporting Hypothesis 4. All core hypothesized paths are supported at the *p* < 0.001 significance level, providing support for the proposed structural relationships.

**Figure 2:**
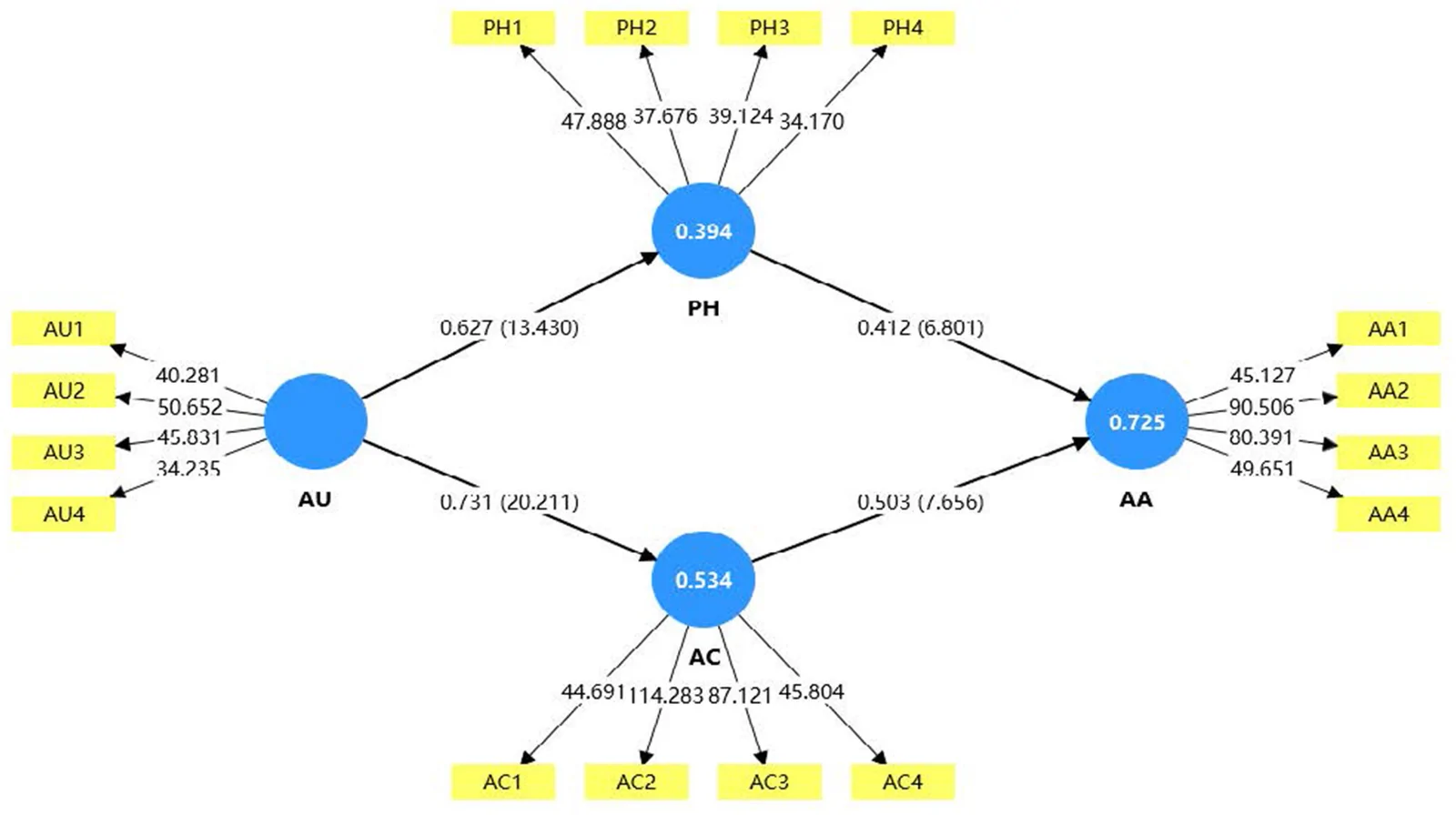
Results of the structural model analysis.

### 5.3 Mediating effects

The mediating roles of perceived honesty (PH) and ability credibility (AC) were examined simultaneously using the bootstrapping procedure in SmartPLS 4.0, with 95% bias-corrected confidence intervals (CIs). A mediating role is supported when the confidence interval of the specific indirect effect does not include zero (Ma et al., 2026). As shown in Table 8, the specific indirect association between AI doctor utilization (AU) and AI health advice adherence intention (AA) through PH was significant [β = 0.258, *t* = 5.493, *p* < 0.001, 95% CI = (0.177, 0.359)], supporting H5. Similarly, the specific indirect association between AU and AA through AC was significant [β = 0.367, *t* = 7.079, *p* < 0.001, 95% CI = (0.261, 0.465)], supporting H6. These findings indicate that PH and AC both serve as significant mediators linking AI doctor utilization to AI health advice adherence intention. The direct effect of AI doctor utilization on AI health advice adherence intention was not significant [β = 0.112, SE = 0.064, *t* = 1.761, *p* = 0.078, 95% CI (−0.018, 0.229)], indicating that the effect was fully mediated by perceived honesty and ability credibility.

**Table 8:** Results of mediating effects.

Hypothesized relationship	β	t-statistics	*p* value	Lower CI	Upper CI	Decision
AU -> PH -> AA	0.258	5.493	0.000	0.177	0.359	Supported
AU -> AC -> AA	0.367	7.079	0.000	0.261	0.465	Supported

### 5.4 Moderating effects

To test the moderating role of anxiety sensitivity (AS) in the cognitive–behavioral link between perceived honesty (PH), ability credibility (AC), and AI health advice adherence intention (AA), interaction terms were estimated in SmartPLS 4.0 and their significance was assessed using the bootstrapping procedure. All variables were standardized to avoid multicollinearity. Moderating effects were tested using the two-stage approach in SmartPLS 4. In the first stage, the latent variable scores were estimated for the main-effect model without the interaction term. In the second stage, the interaction terms were created as the products of the latent variable scores of anxiety sensitivity with perceived honesty and with ability credibility, respectively, and the structural model was re-estimated including these interaction terms. The significance of the interaction effects was assessed using bootstrapping with 5,000 resamples.

The results are presented in Table 9. First, the interaction term between anxiety sensitivity and perceived honesty (AS × PH) was significantly negative (β = −0.165, *t* = 2.233, *p* = 0.026), supporting Hypothesis 7. This result suggests that AS negatively moderates the positive relationship between PH and AA: the positive relationship between PH and AA becomes weaker as anxiety sensitivity increases. Second, the interaction term between anxiety sensitivity and ability credibility (AS × AC) was significantly positive (β = 0.171, *t* = 1.988, *p* = 0.047), supporting Hypothesis 8. This finding indicates that AS positively moderates the positive relationship between AC and AA: the positive relationship between AC and AA becomes stronger as anxiety sensitivity increases. In summary, anxiety sensitivity acts as a dual-directional moderator. It weakens the positive relationship between perceived honesty and AI health advice adherence intention while strengthening the positive relationship between ability credibility and AI health advice adherence intention. These moderating effects are further illustrated in the slope test plots (Figures 3, 4), which show the direction and strength of the moderating patterns. Although the AS × AC interaction reaches the conventional significance threshold (β = 0.171, *p* = 0.047), this effect should be interpreted cautiously given its relatively modest magnitude and proximity to the 0.05 threshold (Ma et al., 2024, 2026). Therefore, the positive moderation of AS on the AC–AA relationship should be regarded as preliminary evidence that requires replication in larger and independent samples. Nevertheless, together with the negative AS × PH interaction, the result is consistent with the proposed asymmetric moderating pattern. Importantly, the interaction effects of AS × PH (*f*^2^ = 0.042) and AS × AC (*f*^2^ = 0.041) were both small in magnitude. Therefore, although the interaction effects provide evidence consistent with the proposed moderating pattern, their substantive magnitude should be interpreted cautiously.

**Table 9:** Moderating effects results.

Hypothesized relationship	Path coefficient	*t* statistics	*p* values	Lower CI	Upper CI	Decision
AS × PH → AA	−0.165	2.233	0.026	−0.325	−0.035	Supported
AS × AC → AA	0.171	1.988	0.047	0.022	0.352	Supported

**Figure 3:**
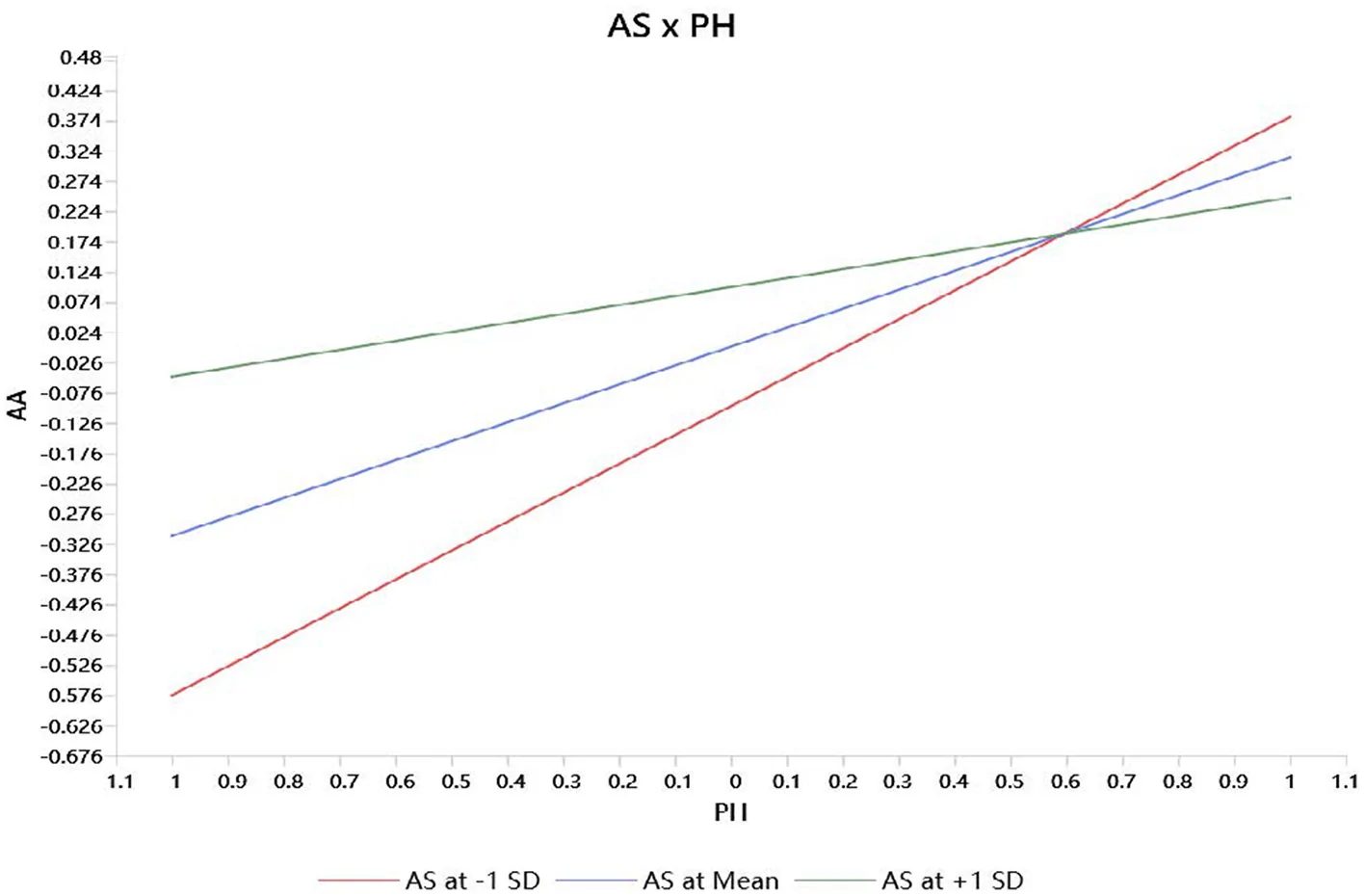
Moderating effect of AS on the PH–AA relationship.

**Figure 4:**
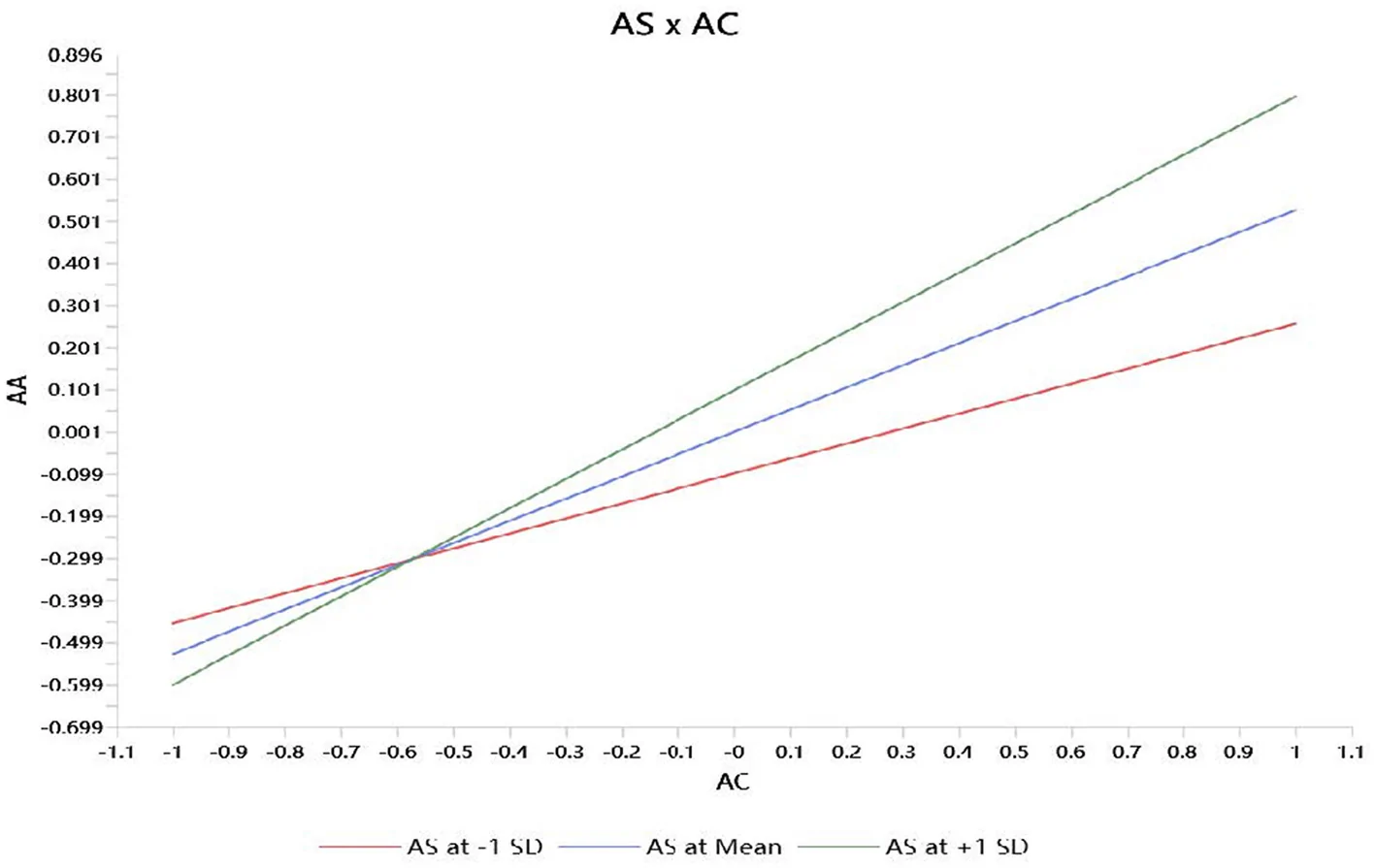
Moderating effect of AS on the AC–AA relationship.

### 5.5 Robustness check for alternative structural specification

To assess whether an alternative structural specification was equally consistent with the observed data, we estimated an alternative model in which perceived honesty (PH) and ability credibility (AC) were specified as antecedents of AI doctor utilization (AU), which in turn was associated with AI health advice adherence intention (AA). The alternative model showed weaker empirical performance than the hypothesized model. Specifically, the explained variance in AA decreased from 0.725 in the hypothesized model to 0.458 in the alternative model. In addition, the estimated-model SRMR increased from 0.067 to 0.140, while the NFI decreased to 0.849. In the alternative model, the standardized path coefficients were 0.584 for AC → AU, 0.201 for PH → AU, and 0.676 for AU → AA, with AU explaining 55.3% of its variance. Taken together, these results indicate that the hypothesized structural specification exhibits better empirical performance than the alternative specification in the present sample. Importantly, however, this comparison should not be interpreted as establishing temporal or causal ordering. Given the cross-sectional nature of the data, reverse or reciprocal relationships between AI doctor utilization and credibility judgments cannot be ruled out. The proposed AU → credibility → adherence ordering is therefore theoretically motivated and empirically more consistent with the present data, rather than causally demonstrated. Longitudinal or experimental research is needed to establish temporal precedence.

## 6 Conclusions

### 6.1 Key findings

First, AI doctor utilization is positively associated with both perceived honesty (PH) and ability credibility (AC), and both credibility judgments are positively associated with AI health advice adherence intention (AA). These findings suggest that users do not evaluate AI doctors through a single, undifferentiated perception of trustworthiness. Rather, greater utilization is associated with two qualitatively different credibility judgments: an honesty-based ethical judgment, concerning whether AI-generated health information is sincere, transparent, and non-deceptive, and a competence-based technical judgment, concerning whether the AI system is accurate, capable, and reliable (Jin et al., 2025; Qin et al., 2024). This distinction is particularly relevant in AI healthcare because users must evaluate not only whether an AI system appears trustworthy in its intentions but also whether it possesses sufficient technical competence to provide reliable health-related guidance. Thus, the findings extend the credibility perspective by showing that users' evaluations of AI doctors involve distinct ethical and technical bases of credibility rather than a generalized trust judgment.

Second, the mediation analyses further show that both perceived honesty (PH) and ability credibility (AC) serve as significant pathways linking AI doctor utilization to AI health advice adherence intention. When PH and AC are simultaneously included in the structural model, the specific indirect association through PH is significant (β = 0.258, *t* = 5.493, *p* < 0.001), as is the specific indirect association through AC (β = 0.367, *t* = 7.079, *p* < 0.001). The direct effect is not significant, indicating that the relationship is fully mediated by perceived honesty and ability credibility. These findings are consistent with prior research highlighting the important role of trust and credibility in shaping users' responses to AI-enabled healthcare services. However, rather than treating credibility as a generalized evaluation, the present study further distinguishes between an honesty-based pathway and a competence-based pathway. This distinction is particularly relevant in the high-stakes context of AI healthcare, where users must consider not only whether AI-generated information is honest and transparent, but also whether it is technically competent, accurate, and reliable. The indirect association through AC is numerically larger than that through PH; however, because the two indirect effects were not formally compared, this difference should not be interpreted as evidence that the AC pathway is statistically stronger. Taken together, the findings suggest that users' adherence intentions may be associated with multiple credibility judgments rather than a single trust-based mechanism, while other factors, such as prior digital health experiences, health beliefs, and social influences, may also contribute to users' responses to AI-generated health advice.

Third, the asymmetric moderating role of anxiety sensitivity (AS) provides an important insight into how credibility judgments are associated with users' AI health advice adherence intention. AS negatively moderates the PH–AA relationship but positively moderates the AC–AA relationship. Specifically, as anxiety sensitivity increases, honesty-based credibility becomes less strongly associated with adherence intention, whereas competence-based credibility becomes more strongly associated with adherence intention. From an information-processing perspective, this asymmetric pattern is consistent with a differentiated credibility-processing perspective, suggesting that anxiety sensitivity may differentially condition the relevance of honesty- and competence-based credibility judgments in users' adherence decisions. In health-related contexts, users with higher anxiety sensitivity may rely more strongly on competence-related evaluations when forming adherence intentions, whereas honesty-based evaluations may play a comparatively weaker role. However, because the study does not directly measure information weighting, cue salience, or shifts in cognitive attention, the underlying psychological process cannot be established from the present data. Moreover, given the relatively modest magnitude of the interaction effects, this interpretation should be regarded as preliminary and warrants replication in larger and independent samples.

### 6.2 Theoretical implications

This study makes several theoretical contributions to the literature on AI-enabled healthcare and information processing theory (IPT). First, it extends existing research on AI health advice adherence intention by shifting attention from what factors predict adherence to how users cognitively evaluate AI-generated health advice. Prior research has primarily emphasized individual characteristics, organizational governance, technical infrastructure, and external environmental factors, with relatively limited attention to the cognitive processes associated with users' interactions with AI healthcare tools (Yang et al., 2026). Drawing on IPT, this study conceptualizes perceived honesty (PH) and ability credibility (AC) as two distinct credibility judgments through which users evaluate the ethical and technical attributes of AI healthcare systems. Rather than claiming to test the entire information-processing sequence of attention, encoding, inference, and response, this study focuses specifically on the credibility inference process, showing how AI doctor utilization is associated with differentiated credibility judgments that, in turn, relate to users' adherence to AI-generated health advice. In doing so, the study extends the application of IPT from a generic stimulus–cognition–behavior framework toward a more context-sensitive explanation of credibility evaluation in AI-enabled healthcare, where diagnostic uncertainty, health risks, and potentially consequential outcomes make the distinction between ethical and technical credibility particularly salient.

Second, this study advances the understanding of the cognitive pathways linking AI doctor utilization to AI health advice adherence intention by revealing a dual credibility mechanism. Existing studies have often conceptualized trust as a general or unidimensional mechanism underlying responses to healthcare technologies. In contrast, our findings show that PH and AC represent distinct mediating pathways between AI doctor utilization and AI health advice adherence intention (Oberoi et al., 2025). This distinction is theoretically important because honesty-based credibility reflects users' evaluations of whether an AI system is sincere, transparent, and non-deceptive, whereas ability-based credibility reflects evaluations of its competence, accuracy, and reliability. Thus, users' adherence to AI-generated health advice cannot be fully understood through a generalized notion of trust. Instead, the findings suggest that users differentiate between ethical credibility and competence-based credibility when evaluating AI healthcare systems. This dual-pathway perspective provides a more fine-grained explanation of how credibility judgments are associated with adherence decisions in high-stakes healthcare contexts.

Finally, this study identifies anxiety sensitivity (AS) as a theoretically meaningful boundary condition in the relationship between credibility judgments and AI health advice adherence intention. Prior research has primarily examined anxiety sensitivity as an emotional characteristic associated with individual cognition and health-related behavior, while its role in conditioning different credibility–behavior relationships has received limited attention. Our findings reveal an asymmetric moderating pattern: as anxiety sensitivity increases, the positive relationship between PH and AI health advice adherence intention weakens, whereas the relationship between AC and adherence intention strengthens. This pattern is consistent with a differentiated credibility-processing perspective, suggesting that the relevance of honesty- and competence-based credibility judgments may vary across levels of anxiety sensitivity. Accordingly, the study extends the application of IPT by showing that individual emotional characteristics can serve as boundary conditions for the relationships between different cognitive evaluations and behavioral intentions. Importantly, because attention allocation, cue salience, and information weighting were not directly measured, the present findings should not be interpreted as evidence of an underlying psychological reweighting process. Future experimental research should directly examine whether anxiety sensitivity changes how users attend to and weight different credibility cues in AI-enabled health decision-making.

### 6.3 Practical implications

This study offers several practical implications for healthcare institutions, AI healthcare developers, and medical service managers seeking to promote appropriate reliance on AI-generated health advice rather than indiscriminate adherence. First, the findings suggest that effective AI healthcare services should simultaneously strengthen competence-based and honesty-based credibility. Because AI doctor utilization is associated with AI health advice adherence intention through both perceived honesty and ability credibility, encouraging the use of AI healthcare tools alone may be insufficient; what matters is whether users develop appropriate credibility judgments through their interactions with these systems. Healthcare institutions should therefore integrate AI tools into appropriate healthcare and health-management scenarios while providing users with sufficient guidance on how to interpret and evaluate AI-generated recommendations. Rather than presenting AI as an autonomous substitute for professional medical judgment, institutions should position it as a decision-support resource whose recommendations should be evaluated in light of their accuracy, limitations, and clinical context.

Second, healthcare institutions and AI developers should strengthen both the technical reliability and transparency of AI healthcare systems. To enhance ability credibility, developers should prioritize the accuracy, consistency, and reliability of AI-generated health information, continuously update medical knowledge bases, and establish appropriate validation and quality-control mechanisms. Particularly for health-related recommendations with potentially serious consequences, AI systems should communicate uncertainty and clearly indicate situations in which professional medical consultation is necessary. At the same time, perceived honesty can be supported through transparent information provision. Users should be informed about the sources, reasoning basis, limitations, and uncertainty of AI-generated recommendations, while potentially misleading, overly confident, or ambiguous outputs should be minimized. Healthcare institutions can further support these efforts by communicating how AI systems are validated, monitored, and incorporated into healthcare services (Yuan and Lee, 2026). Together, these practices can help users develop calibrated credibility judgments, rather than simply increasing their general trust in AI.

Third, the asymmetric moderating role of anxiety sensitivity suggests that a one-size-fits-all credibility strategy may be ineffective in AI healthcare. As anxiety sensitivity increases, the role of PH in AI health advice adherence intention weakens, whereas the role of AC strengthens. Healthcare providers and AI developers should therefore consider users' psychological differences when designing credibility communication strategies. For users who are particularly sensitive to health-related threats, emphasizing only reassuring or honesty-related messages may be insufficient. Instead, AI systems should provide clear evidence of technical reliability, explain the basis and uncertainty of recommendations, and explicitly communicate the boundaries of AI capabilities. For less anxiety-sensitive users, transparent communication regarding how information is generated, what the system can and cannot do, and how user interests are protected may remain particularly valuable. Such differentiated communication can help align credibility cues with users' information-processing needs without encouraging excessive dependence on AI-generated advice.

Finally, the findings have implications for human–AI collaborative healthcare governance. For low-risk and routine health inquiries, AI systems may provide accessible informational and decision support. For high-risk, uncertain, or potentially life-critical situations, however, AI-generated recommendations should incorporate clear escalation mechanisms that direct users toward qualified healthcare professionals. Healthcare institutions can accordingly develop tiered human–AI collaboration protocols in which the degree of human oversight increases with the potential consequences and uncertainty of AI-generated recommendations. In this way, the practical objective should not be to maximize AI health advice adherence intention, but to foster appropriate and calibrated reliance—encouraging users to rely on AI when its recommendations are sufficiently credible while recognizing when human medical judgment remains necessary.

### 6.4 Limitations and future research

This study has several limitations that provide directions for future research. First, the cross-sectional design limits causal inference regarding the relationships among AI doctor utilization, credibility judgments, and AI health advice adherence intention. Reverse relationships are also possible, as users who perceive AI as more credible may be more likely to use AI doctors. Although the final sample of 224 respondents exceeded the minimum sample size indicated by the statistical power analysis, a larger sample would provide greater statistical precision, particularly for detecting relatively small interaction associations, and would strengthen the replicability and stability of the findings. Future research could therefore employ larger independent samples, longitudinal designs, or experimental approaches to establish temporal ordering and assess the robustness of the proposed relationships (Luo et al., 2026; Qian et al., 2025). Second, the generalizability of the findings is constrained by the characteristics of the sample. Although participants were recruited from several regions of China, the sample was concentrated primarily in Shandong Province and consisted of general public users with prior experience using AI-based health-advice chatbots rather than clinical patient populations. In addition, only five participants were under 18 years of age, limiting the relevance of the findings to younger users. Therefore, the present results should not be generalized to clinical adherence behavior or specific patient populations. Future research should replicate the model using geographically diverse samples and clinically relevant populations and examine actual adherence behavior in specific healthcare contexts. Analytical frameworks developed in adjacent scientific fields for decomposing complex, multi-stage processes and their underlying mechanisms (Gan et al., 2026) may serve as methodological templates for modeling the sequential pathways through which users repeatedly evaluate and act on AI-generated health advice. Third, the study relies on self-reported survey measures, and AI health advice adherence intention primarily reflects users' reported willingness or tendency to follow AI-generated advice rather than directly observed behavior. Future research could incorporate behavioral or multi-wave data to validate actual adherence. Moreover, the relative importance of honesty- and competence-based credibility may vary across AI applications, medical risk levels, and human–AI collaboration arrangements. Examining these contexts would help clarify whether the observed pattern of differentiated credibility associations is robust across different AI healthcare contexts. Nevertheless, because all focal variables were collected from the same respondents using self-reported measures at a single point in time, common method variance cannot be completely ruled out despite the statistical diagnostics and procedural remedies. Future research could employ multi-source or temporally separated data collection designs. Future research should attend to the additional, and often overlooked, dimensions of AI governance (Chen, 2026) when designing data-collection procedures in sensitive health contexts.

## Data Availability

The raw data supporting the conclusions of this article will be made available by the authors, without undue reservation.
